# Review of Stress and Animal Welfare by Donald Broom and Ken G. Johnson

**DOI:** 10.3390/ani10020363

**Published:** 2020-02-24

**Authors:** Temple Grandin

**Affiliations:** Department of Animal Science, Colorado State University, Fort Collins, CO 80523, USA; Cheryl.Miller@ColoState.EDU

In the first chapter [1], one of the most fundamental concepts on understanding stress is presented. It is too simplistic to assume that every rise in cortisol or other physiological indicators of stress is harmful and an indication of poor welfare. Cortisol will be elevated when a painful procedure such as castration is performed, but it is also elevated during positive experiences such as courtship or mating. The authors define stress that is detrimental to animal welfare with the following statement—“Stress is an environmental effect on the individual which overtaxes its control systems and reduces its fitness or appears to do so.” To state this in a slightly different way, stress is the negative consequence of forcing the control systems in an animal “to work too hard for effective functioning, that is to overtax them.” The authors also use an athletic analogy. When a runner becomes exhausted, was it “stressful or was it training or was it both of these?” This reviewer asks—was running something the animal or person wanted to do?

Another important basic idea which is presented in two chapters is the difference between long-term responses to the environment and short-term responses. Painful procedures such as castration or non-painful stressful procedures such as restraint are both short-term. Castration definitely causes pain, but restraint is not painful, although it may be highly aversive. An example of long-term welfare issues may be housing that causes injury or greatly restricts movement or natural behaviors. The two chapters outline methods to evaluate both short-term and long-term welfare issues. Another important behavioral fact that was outlined in Chapter 5 was in the discussion of the assessment of pain. How an animal behaves when it is in pain differs depending on how it would react in the wild to avoid predators. Rabbits are inactive when they are in pain and dogs or pigs will be more active. When living in the wild, a rabbit in pain may have a better chance at survival if it stays still.

This book is an excellent reference text for both students who will be studying animal welfare and researchers in the field. It provides an excellent background on both physiological and behavioral measures. This will be especially useful for people entering into research on animal welfare. It emphasizes the need to use more than one behavioral measure. This is especially important with some new measurement tools such as Qualitative Behavioral Analysis (QBA). This method can be affected by observer biases.

The last chapter covers Ethics and Issues in the World such as sustainability and food production systems in the future. How different people view some of these issues often depends on their value systems. The authors state that the objective scientific measures that are outlined in the book can provide part of the answers on the choice of farm practices. A defining factor may be the attitude of the public on what is ethically acceptable. In the final paragraph, Broom and Johnson present many questions. If animal production was totally stopped, then there would be many lands (grazing range) which would stop producing food. This may cause more intensive farming on the land that remains because grazing land cannot be cropped. People also forget that animals are killed during crop production. In a final statement, the authors describe a sustainable system where ruminant grazing is part of the system.

It is really important for readers to have a source that preserves older classic studies. This book does a good job of this. The only weakness is that a few more new references on some of the behavioral measures would have been a great addition. This book is recommended for people who will be doing research in animal welfare because it provides an overview of most of the methods for measuring physiology and behavior. It will be especially useful for new research scientists in animal welfare.
